# Slow Bursting Neurons of Mouse Cortical Layer 6b Are Depolarized by Hypocretin/Orexin and Major Transmitters of Arousal

**DOI:** 10.3389/fneur.2016.00088

**Published:** 2016-06-15

**Authors:** Anne-Laure Wenger Combremont, Laurence Bayer, Anouk Dupré, Michel Mühlethaler, Mauro Serafin

**Affiliations:** ^1^Département des neurosciences fondamentales, Centre Médical Universitaire, Geneva, Switzerland; ^2^Centre de médecine du sommeil, Hôpitaux Universitaires de Genève, Geneva, Switzerland

**Keywords:** noradrenaline, acetylcholine, neurotensin, dopamine, histamine, sleep, arousal

## Abstract

Neurons firing spontaneously in bursts in the absence of synaptic transmission have been previously recorded in different layers of cortical brain slices. It has been suggested that such neurons could contribute to the generation of alternating UP and DOWN states, a pattern of activity seen during slow-wave sleep. Here, we show that in layer 6b (L6b), known from our previous studies to contain neurons highly responsive to the wake-promoting transmitter hypocretin/orexin (hcrt/orx), there is a set of neurons, endowed with distinct intrinsic properties, which displayed a strong propensity to fire spontaneously in rhythmic bursts. In response to small depolarizing steps, they responded with a delayed firing of action potentials which, upon higher depolarizing steps, invariably inactivated and were followed by a depolarized plateau potential and a depolarizing afterpotential. These cells also displayed a strong hyperpolarization-activated rectification compatible with the presence of an *I*_h_ current. Most L6b neurons with such properties were able to fire spontaneously in bursts. Their bursting activity was of intrinsic origin as it persisted not only in presence of blockers of ionotropic glutamatergic and GABAergic receptors but also in a condition of complete synaptic blockade. However, a small number of these neurons displayed a mix of intrinsic bursting and synaptically driven recurrent UP and DOWN states. Most of the bursting L6b neurons were depolarized and excited by hcrt/orx through a direct postsynaptic mechanism that led to tonic firing and eventually inactivation. Similarly, they were directly excited by noradrenaline, histamine, dopamine, and neurotensin. Finally, the intracellular injection of these cells with dye and their subsequent Neurolucida reconstruction indicated that they were spiny non-pyramidal neurons. These results lead us to suggest that the propensity for slow rhythmic bursting of this set of L6b neurons could be directly impeded by hcrt/orx and other wake-promoting transmitters.

## Introduction

Neocortical neurons with a rhythmic bursting pattern of activity have been reported in many *in vitro* studies (1–7). This kind of activity has been linked to the UP and DOWN states recorded from neocortical neurons *in vivo* [for reviews, see Ref. (8, 9)] as well as the slow oscillations (SO) (10–12), which occur during slow-wave sleep (SWS). The mechanism of the cortical SO is not fully understood (8, 9, 13–15), but the discovery, in cortical layers 2–3 and 5, of cells with rhythmic activities in the absence of synaptic transmission (7, 16, 17) has led to the suggestion that some scattered “pacemaker” cells could contribute to cortical rhythmic activities in conjunction with recurrent excitatory circuits (8). This suggestion has been recently reinforced by the demonstration that optogenetic activation of a small number of infragranular neurons *in vivo* can initiate slow oscillatory network activity (18).

In studies of L6b in rat (19) and mouse (20) brain slices of the primary somatosensory cortex, we have shown that it is the only cortical layer in which neurons exhibit a direct postsynaptic response to hypocretin/orexin (hcrt/orx), a prominent peptidergic wake-promoting transmitter [for recent reviews on hcrt/orx, see Ref. (21–26)]. The L6b is, in many species (27–31), the remnant into adulthood of the subplate, a layer known for its transient role during early development [for review, see Ref. (32)]. We have previously argued (19) that this layer, through projections to superficial cortical layers (27, 28), could relay the arousing action of hcrt/orx.

As a substrate of SO during SWS, neurons and networks contributing to UP and DOWN states should be sensitive to the action of wake-promoting transmitters (8, 33, 34), which, at the transition from sleep to waking, would impede their oscillatory activity. The goal of this study was thus to investigate the presence of spontaneous slow rhythmic firing cells in L6b of mouse cortical brain slices and determine whether they are sensitive to hcrt/orx and other transmitters of arousal. Early results have been presented in a preliminary form (35).

## Materials and Methods

### Slice Preparation

Brain slices were obtained from 17- to 21-day-old juvenile C57Bl/6 mice (Charles River Laboratories, France). Mice, treated according to the rules of the Swiss Federal Veterinary Office (approval ID: 31.1.1007/3248/0), were anesthetized by Isoflurane (Forene, Abbott AG, Baar, Switzerland), decapitated, and the brain was extracted. Coronal sections (300 μm thick), at the level of the primary somatosensory cortex (SSp), were then cut on a vibrating blade microtome (Leica VT1200s, Leica Biosystems, Nussloch, Germany) into a cold slicing medium (36) containing (in millimolars): 135 *N*-methyl-d-glucamine (NMDG), 0.5 CaCl_2_, 20 choline bicarbonate, 10 glucose, 1 KCl, 1.2 KH_2_PO_4_, and 1.5 MgCl_2_ (NMDG was first dissolved and then titrated to pH 7.4 with HCl). Following the slicing, tissue was left to recover at 37°C for 1 h and then kept at room temperature (RT), until use, in artificial cerebrospinal fluid (ACSF) containing (in millimolars): 2.4 CaCl_2_, 10 glucose, 5 KCl, 1.25 KH_2_PO_4_, 130 NaCl, 20 NaHCO_3_, and 1.3 MgSO_4_ aerated with carbogen (95% O_2_ and 5% CO_2_). During the recovery period and the time left at RT, 1 mM of kynurenic acid was added to the ACSF. Individual slices were then transferred to a thermoregulated (32°C) recording chamber placed on the stage of an upright microscope (Axioskop, Zeiss, Oberkochen, Germany) equipped with an infrared camera (TILL-Photonics). Slices were maintained, immersed, and were continuously superfused (4–5 ml/min) with ACSF bubbled with 95% O_2_ and 5% CO_2_.

### Electrophysiological Recordings

**Figure 1:**
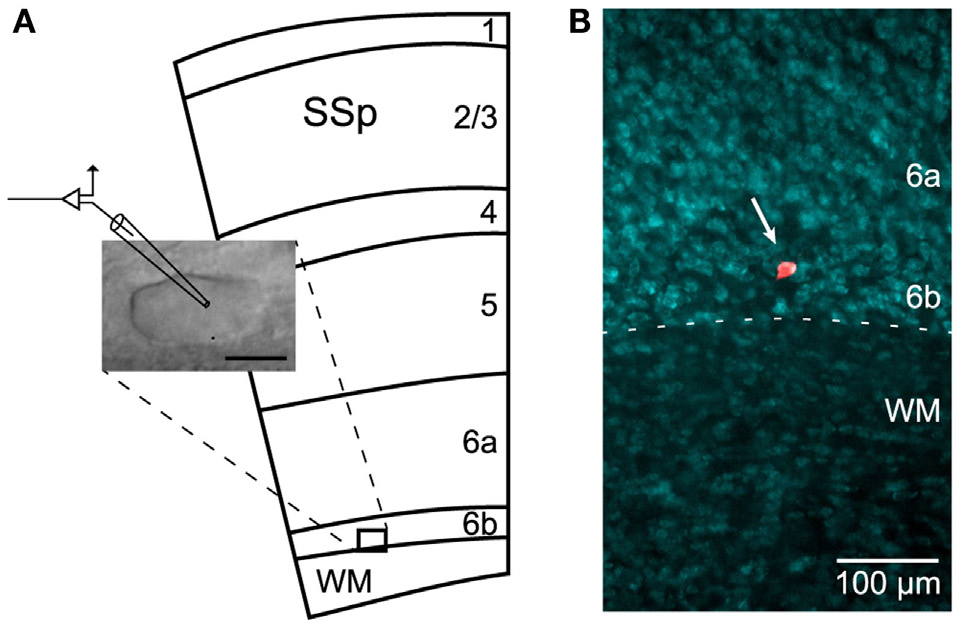
**Recordings in L6b**. **(A)** Schematic illustration (adapted from the Allen Mouse Brain Atlas; http://mouse.brain-map.org) of the primary somatosensory cortex (SSp) and underlying white matter (WM), together with image of a recorded neuron. Scale bar: 10 μm. **(B)** Localization of a dye-injected (Alexa Fluor) cell in layer 6b (arrow) using fluorescent Nissl counterstaining.

Recordings were performed on cells located in cortical L6b (Figure 1A). This layer is defined as a thin and compact band of neurons lying immediately above the white matter (WM) of the corpus callosum, in the deepest part of the gray matter, below the layer 6a (27, 29, 36). In our experiments, such a compact cell layer could be identified under infrared microscopy and neurons therein were preferably recorded the closest to the WM/L6b boundary.

The recordings were performed in the whole-cell configuration in current-clamp mode using an Axopatch 200A amplifier (Molecular Devices, Sunnyvale, CA, USA) and pClamp 10.0 software (Molecular Devices, Sunnyvale, CA, USA). Patch pipettes (7–12 MΩ) were pulled on a DMZ universal puller (Zeitz-Instruments, Munich, Germany) from borosilicate glass capillaries (GC150F-10; Harvard Apparatus, Holliston, MA, USA) and filled with an intrapipette solution containing (in millimolars): 3 ATP, 0.1 BAPTA, 0.1 GTP, 10 HEPES, 4 KCl, 126 KMeSO_4_, 5 MgCl_2_, and 8 phosphocreatine disodium salt, pH 7.3 (285–300 mOsm). In order to allow for a subsequent morphological analysis, Alexa Fluor 555 Hydrazide (200 μM; Molecular Probes by Life Technologies, Carlsbad, CA, USA) was occasionally added to the intrapipette solution. Membrane potential values were measured immediately after the realization of the whole-cell configuration and were not compensated for junction potential [estimated at −9.6 mV (37)]. In several experiments, synaptic transmission was blocked by either adding TTX (10^−6^M) to the ACSF or increasing its magnesium and lowering its calcium concentration (10 mM Mg^2+^ and 0.1 mM Ca^2+^, respectively).

### Chemicals

The peptide hypocretin1/orexinA (hcrt1/orxA) (Bachem, Bubendorf, Switzerland) was used exclusively in this study since it is known to have a relatively similar affinity for OX1 and OX2 receptors, whereas hypocretin2/orexinB (hcrt2/orxB) has a lower affinity for OX1 receptors than hcrt1/orxA (38). For brevity, hcrt1/orxA will be referred to as hcrt/orx. Noradrenaline (NA), histamine, dopamine, neurotensin (NT), DNQX, and NBQX were all obtained from Sigma-Aldrich (Buchs, Switzerland), whereas AP5 and bicuculline were obtained from Tocris Bioscience (Bristol, UK) and tetrodotoxin (TTX) from Latoxan (Valence, France). All the drugs were bath applied.

### Histology

Following electrophysiological recordings, a subset of slices with only 1 or 2 dye-injected cells were fixed in a solution of 4% paraformaldehyde for 1–2 h. Slices were then counterstained using NeuroTrace 435/455 blue-fluorescent Nissl stain (Molecular Probes by Life Technologies, Carlsbad, CA, USA) to confirm the location of Alexa Fluor-filled neurons in L6b (Figure 1B). For that purpose, after washing in PBS, the slices were incubated twice for 2 h at RT, first in PBS-Triton 0.3% and then in NeuroTrace 1/100 in PBS-Triton 0.3%. After an overnight washing step in PBS, the slices were mounted in FluorSave (Calbiochem) and coverslipped. Images were acquired on an inverted epifluorescence microscope (Nikon Eclipse Ti; Nikon Instruments, Melville, NY, USA) using a 10× magnification objective.

### Neurolucida Reconstruction and Morphometric Analysis

Alexa Fluor-filled neurons were imaged using a confocal laser scanning microscope (Zeiss LSM700; Zeiss, Oberkochen, Germany). Confocal image stacks were acquired at three different magnifications (20×, 40×, and 63×) to cover the entire dendritic arborization. Dendritic and axonal trees were next reconstructed in 3D from the confocal stacks using Neurolucida 11 software (MBF Bioscience, Williston, VT, USA). Once the tracing was completed, several somatodendritic and axonal features were analyzed using Neurolucida Explorer 11 software (MBF Bioscience, Williston, VT, USA). The reconstructions of neurons were aligned to have the WM/L6b boundary parallel to the *x*-axis. Only neurons with minimal extracellular dye leakage and with a total axonal length above 1.5 mm were considered. Five somatic parameters were quantified: the cell body perimeter and its area, the maximal and minimal ferets, as well as the aspect ratio. Maximal and minimal ferets were defined, respectively, as the longest and smallest diameters of the soma, measured between two parallel tangential lines drawn along the soma boundaries (as if measured with a caliper). The aspect ratio, defined by the ratio of the maximal feret to the minimal feret, gives an indication about the elongation of the cell body. In order to investigate the dendritic arborization, the following parameters were quantified: the number of primary dendrites, the total dendritic length, the number of nodes (branching points), the dendritic branching frequency (i.e., number of nodes/100 μm), and the ratio of the total dendritic length to the total dendritic surface. In order to further analyze the spatial distribution of the dendrites, a Sholl analysis and a polar histogram were also performed. The Sholl analysis, used to represent the dendritic density around the cell body, was expressed as the fraction of the total dendritic length contained in a series of spheres centered at the centroid of the soma with incremental radii (100, 200, and 300 μm). The polar histogram, on the other hand, describes the overall orientation of the dendritic processes in which their 3D reconstruction was reduced to a 2D polar histogram with dendritic lengths plotted, in bins, as a function of direction. The dendritic arborization around the soma was indeed divided in 120 bins of 3° with cubic spline smoothing for each dendritic reconstruction. The average of the dendritic reconstructions of the whole set of cells was calculated and a mean polar plot was obtained using Statgraphics Centurion XVII (Statpoint Technologies, Warrenton, VA, USA) in which the 0°–180° axis was parallel to the WM/L6b boundary. On the polar plot, processes pointing to 90° were oriented toward the pial surface whereas those directed to 270° were oriented toward the WM. In order to characterize the axonal arborization and its spatial distribution, a procedure similar to the one outlined above for the dendritic trees, was applied. Each parameter is presented as mean ± SEM.

## Results

Whole-cell current-clamp recordings were performed on L6b neurons of the mouse primary somatosensory cortex (Figures 1A,B).

### Intrinsic Properties of a Class of L6b Neurons with a Propensity for Slow Bursting Activity

In the process of looking for slow spontaneous rhythmic activity in L6b, we found a class of cells having specific intrinsic membrane properties and a propensity for slow rhythmic burst firing.

**Figure 2:**
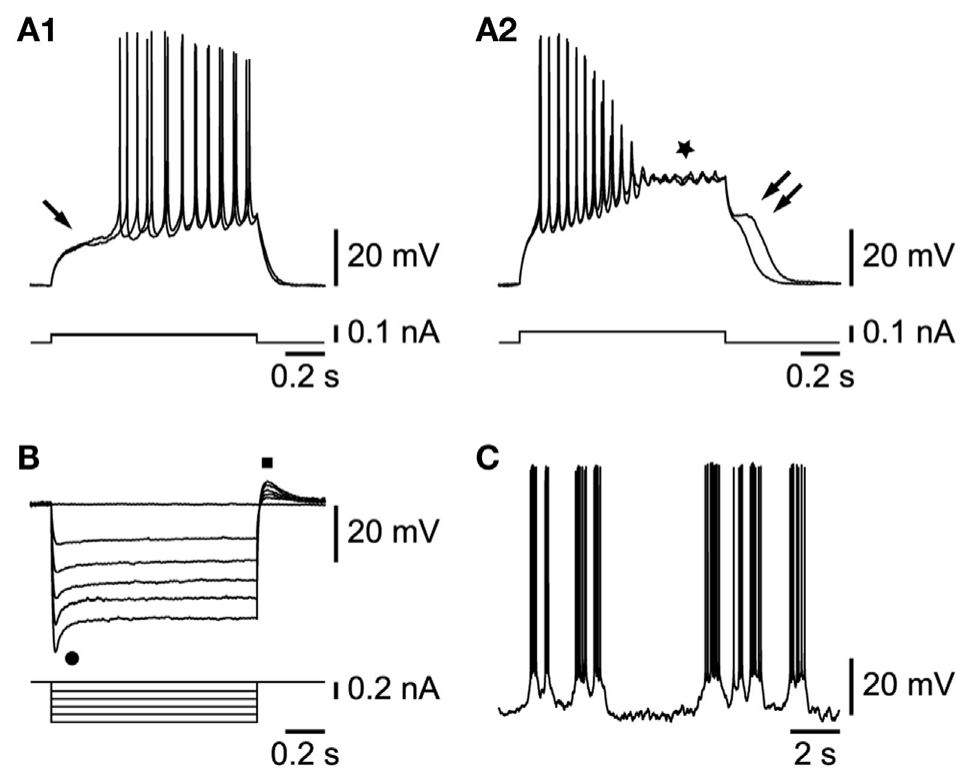
**Properties of L6b slow bursting neurons**. **(A1)** Membrane depolarization in response to small current steps (arrow indicates delayed firing). **(A2)** Increasing current step injection leads to action potential inactivation, a plateau potential (star), and a depolarizing afterpotential (double arrow). **(B)** Hyperpolarizing current steps yield voltage- and time-dependent rectification (dot) and rebound (square). **(C)** Rhythmic bursting activity at resting membrane potential.

Cells in this class were characterized first by their response to depolarizing current injections. Beyond their passive responses to small current steps, they displayed a delayed (Figure 2A1, arrow) firing of action potentials, with little accommodation. In response to larger current steps, action potentials always inactivated rapidly and were followed by a plateau potential (Figure 2A2, star) often endowed with subthreshold oscillations. At the break of the current step, a depolarizing afterpotential of a variable duration, signed the voltage-dependent exit from the plateau potential (Figure 2A2, double arrow). Another major characteristic of these cells was revealed by hyperpolarizing pulses. All of these cells were indeed characterized by a strong time- and voltage-dependent inward rectification (Figure 2B, dot), which recovered, at the offset of the current step, in the form of a depolarizing rebound (Figure 2B, square). The blocking of this rectification with 3 mM of cesium chloride (*n* = 2/2, not shown) indicates that it depended on the presence of an *I*_h_ current.

Among the 50 cells endowed with the properties described above, a subset (*n* = 33) could be studied further. As illustrated in Figure 2C, most of them had a propensity for slow rhythmic bursting. They could be subdivided in two sets. The first set, representing the vast majority of cells (30/33), was characterized by neurons that fired in bursts either spontaneously or when hyperpolarized by current injection, whereas a smaller set had cells that displayed an irregular tonic firing (*n* = 3/33) at any membrane potential level.

**Figure 3:**
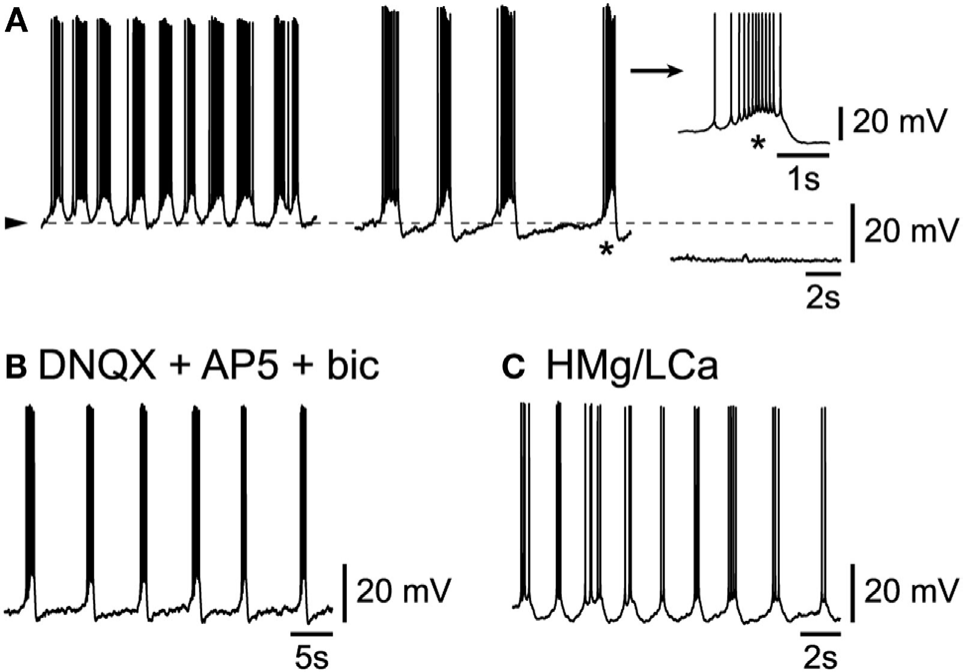
**Intrinsic origin of slow rhythmic burst firing in L6b neurons**. **(A)** Under progressive hyperpolarization, frequency of bursts decreases (middle panel) until firing ceases altogether (right panel). **(B)** Persistence of rhythmic bursting activity in presence of DNQX, AP5, and bicuculline. **(C)** Persistence of rhythmic bursting activity in presence of a high Mg/low Ca ACSF (HMg/LCa).

Among the 30 cells with a propensity for burst firing reported above, 20 were chosen for further analysis because they either fired spontaneously in bursts already at rest (8/20) or when held in a range of voltage membrane potentials between −55 and −60 mV, which corresponds to the range of resting potentials recorded in these cells (−57.8 ± 0.6 mV, *n* = 25). This is particularly important given the voltage dependence of the bursting activity (see next paragraph). Under this condition, we found that 9/20 cells fired bursts in a mostly irregular way (Figure 2C), while 11/20 cells fired in a rather regular pattern (Figure 3A). The irregular firing cells had bursts that varied considerably during their activity and could therefore not be used to provide a reliable measure of firing parameters. In contrast, in 9 out of the 11 regular bursting cells, such measures could be obtained and the mean burst duration varied between 1.3 ± 0.3 and 3.1 ± 0.6 s (range 0.5–6.5 s, *n* = 9), whereas the mean frequency of occurrence was 0.4 ± 0.1 Hz (range 0.1–0.8 Hz, *n* = 9). The firing frequency within the bursts was 15.8 ± 2.0 Hz (range 6.3–30.9 Hz, *n* = 9). Two of the 11 regular firing cells, with bursts of a much higher duration (from 8.5 to 35 s) and much lower frequency of occurrence (from 0.01 to 0.03 Hz), were not included in the analysis.

### Intrinsic Origin of the Slow Bursting Activity of L6b Neurons

A major issue concerning these L6b cells, is whether their spontaneous activity depended solely on their intrinsic properties, in which case they could be considered as “pacemakers” (7, 8, 14), or whether it depended on a synaptic network to which they belong or a mix thereof. In the conditions of this study in L6b, the vast majority (*n* = 25/30) of cells presented an activity that apparently depended solely on their intrinsic properties. First indeed, when L6b slow bursting neurons were submitted to a progressive membrane hyperpolarization, their burst frequency decreased in a voltage-dependent manner (Figure 3A; *n* = 9/9). As evident in the example of Figure 3A (right panel), at the most hyperpolarized level of membrane potential, no sign of an underlying synaptic activity can be seen. Second, when ionotropic glutamatergic receptors were blocked using the AMPA/kainate antagonist DNQX (or NBQX, both at 2.10^−5^M) together with the NMDA antagonist AP5 at 5.10^−5^M, the rhythmic bursting activity of L6b neurons persisted (*n* = 3/3, not shown). As GABAergic-dependent rhythmic activity could persist in a subset of cortical neurons after the complete blocking of ionotropic glutamatergic activity (16), bicuculline (at 10^−5^M), a GABA_A_ antagonist, was applied to cells already in presence of the AMPA/kainate and NMDA antagonists. In this condition, as evident in Figure 3B, rhythmic activity was found to persist (*n* = 2/2). Finally, when cells were submitted to a complete synaptic blocking using a high magnesium/low calcium (high Mg/low Ca) ACSF for prolonged periods of time, they were still able to show rhythmic bursting (Figure 3C; *n* = 3/3).

### Synaptically Driven Rhythmic Activity in a Subset of L6b Neurons

**Figure 4:**
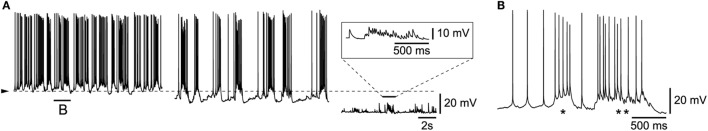
**Mix of intrinsic burst firing and synaptically driven UP and DOWN states**. **(A)** Progressive DC hyperpolarization uncovers underlying rhythmic synaptic events (enlargement in inset). **(B)** Enlargement from part of left panel A with an intrinsic burst (*) and a synaptically driven burst (**).

In contrast to the situation described above, in a small minority (*n* = 5/30) of bursting L6b neurons, spontaneous intrinsic bursts were intermingled with synaptically driven ones. An example of such a situation is illustrated in Figure 4, in which the progressive hyperpolarization of a cell (Figure 4A, from left to right) resulted first in a decreased rate of occurrence of the rhythmic bursts and then, after elimination of the action potentials, in the uncovering of underlying rhythmic bursts of depolarizing synaptic potentials (enlarged in inset of Figure 4A). The mixing of intrinsic and network activity is illustrated in Figure 4B (enlargement obtained from left panel in Figure 4A) which shows that, while one burst appeared to be intrinsically generated [Figure 4B (*)], another contiguous one [Figure 4B (**)], was clearly riding a wave of synaptic activity. It is finally of note that such synaptically driven rhythmic firing was eliminated (*n* = 2/2, not shown) in presence of either TTX or a high Mg/low Ca ACSF. The synaptically driven rhythmic bursts illustrated here, are very similar to the UP states reported previously in other cortical layers (8, 9).

### Responses of L6b Slow Bursting Neurons to Hcrt/Orx and Other Transmitters of Arousal

**Figure 5:**
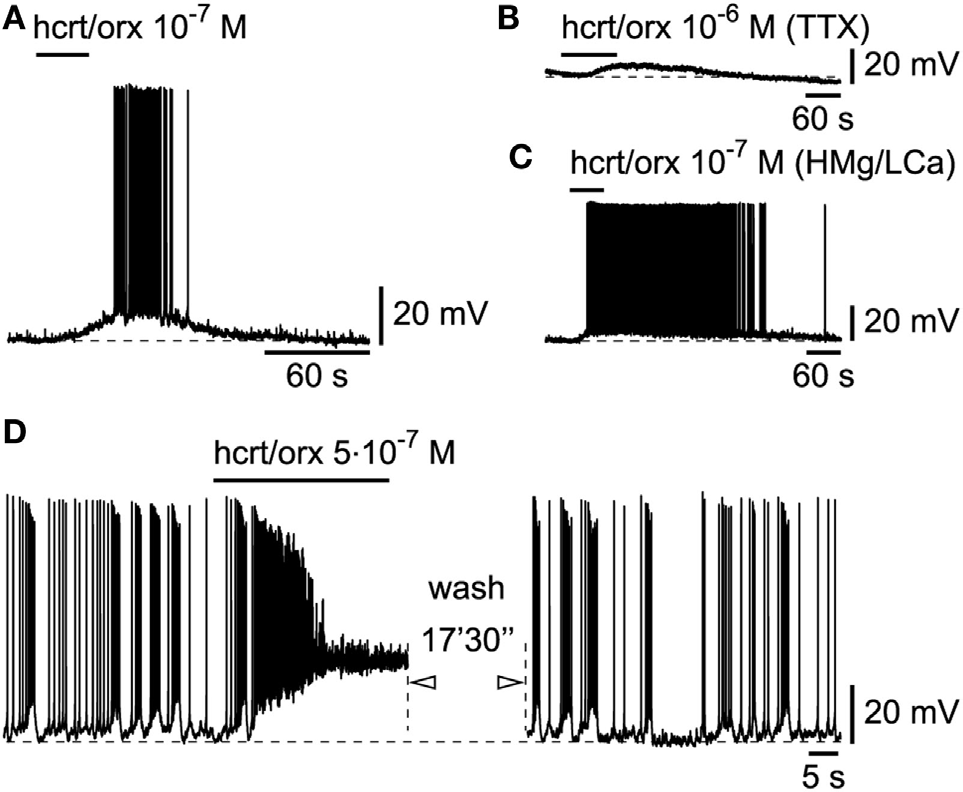
**Hcrt/orx action on L6b slow bursting neurons**. **(A)** Depolarizing and excitatory effect of hypocretin/orexin (hcrt/orx), which persists in presence of TTX **(B)** or synaptic blockade **(C)**. **(D)** Stronger effect of hcrt/orx leads to inactivation of firing (left panel) which recovers later after wash (right panel).

In line with our original publication in rat cortical brain slices (19), we next tested whether the L6b slow bursting neurons were sensitive to hcrt/orx. This was indeed the case as 26/27 tested neurons (Figure 5A) were depolarized and excited by hcrt/orx (applied briefly from 5.10^−8^ to 10^−6^M). As previously reported (19), the neurons were activated through the presence of postsynaptic receptors as they still responded (Figures 5B,C) to hcrt/orx in presence of either TTX (*n* = 2/3) or a high Mg/low Ca ACSF (*n* = 3/4). In these highly excitable cells, application of hcrt/orx often (*n* = 15/26) resulted, even with brief applications, in a complete, but reversible, inactivation of firing (Figure 5D).

**Figure 6:**
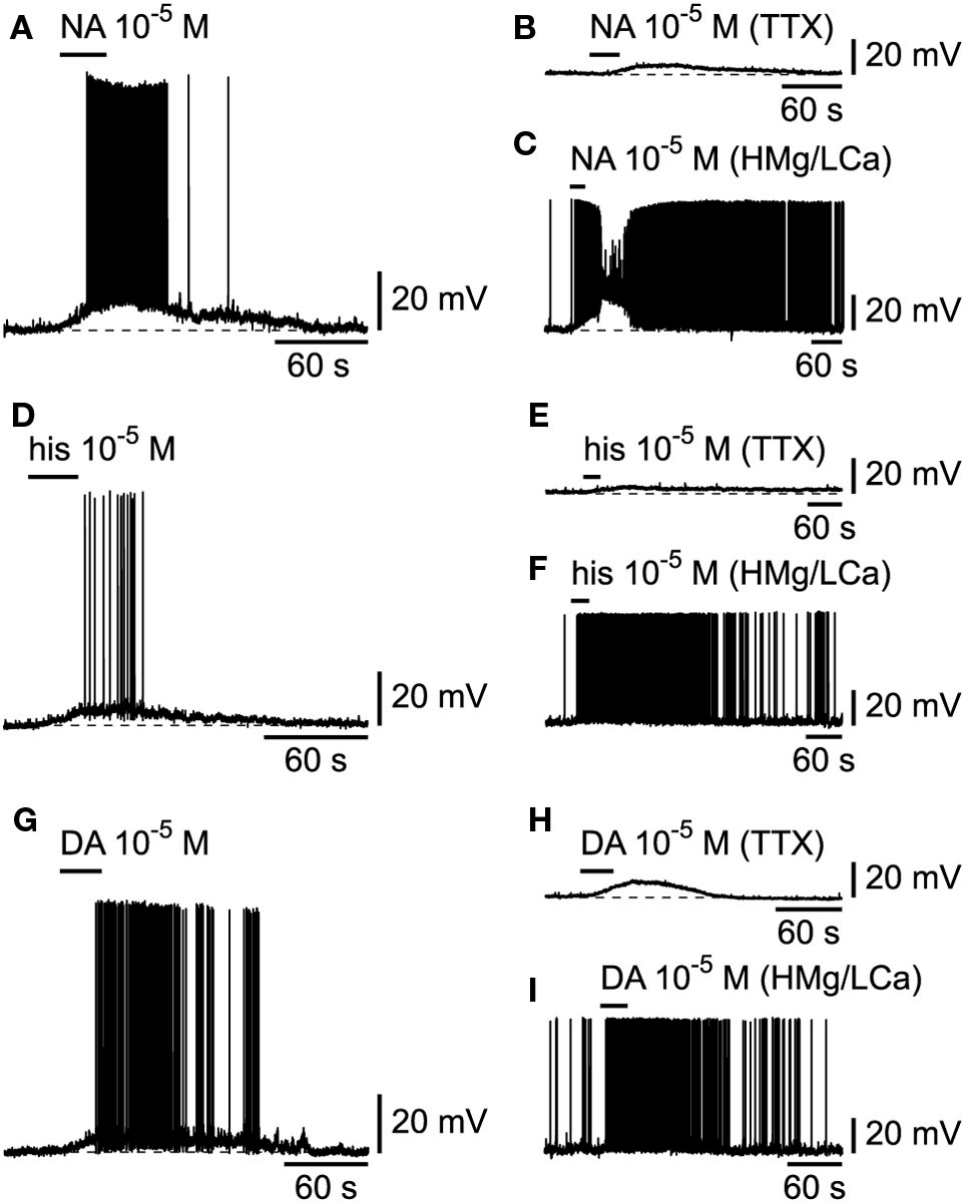
**Effects of aminergic transmitters on L6b slow bursting neurons**. **(A)** Depolarizing and excitatory effect of noradrenaline (NA), which persists in presence of TTX **(B)** or synaptic blockade **(C)**. **(D–F)** Same for histamine (his). **(G–I)** Same for dopamine (DA).

We next tested the effects of other wake-promoting transmitters on cells that had beforehand responded to hcrt/orx. Among them, the aminergic transmitters originating from the brainstem or hypothalamus (NA, dopamine, and histamine) have long been known to play a major role in promoting cortical activation [for review, see Ref. (34)]. We thus first tested NA (10^−5^M) and found that L6b bursting neurons were systematically depolarized and excited (Figure 6A; *n* = 5/5) by this transmitter and that its effect persisted (Figures 6B,C) when applied at 10^−5^M in presence of either TTX (*n* = 2/2) or a high Mg/low Ca ACSF (*n* = 4/4), indicating that it was postsynaptic. Similar depolarizing and excitatory responses were also found for histamine (Figure 6D; *n* = 4/4) applied from 10^−5^ to 10^−4^M, and the effect was again postsynaptic (Figures 6E,F; *n* = 5/5 in presence of either TTX or a high Mg/low Ca ACSF). Similar results were also found for dopamine (*n* = 2/2) applied at 10^−5^M, as illustrated in Figure 6G. Here again, the effect persisted (Figures 6H,I; *n* = 5/5) in presence of either TTX or a high Mg/low Ca ACSF.

**Figure 7:**
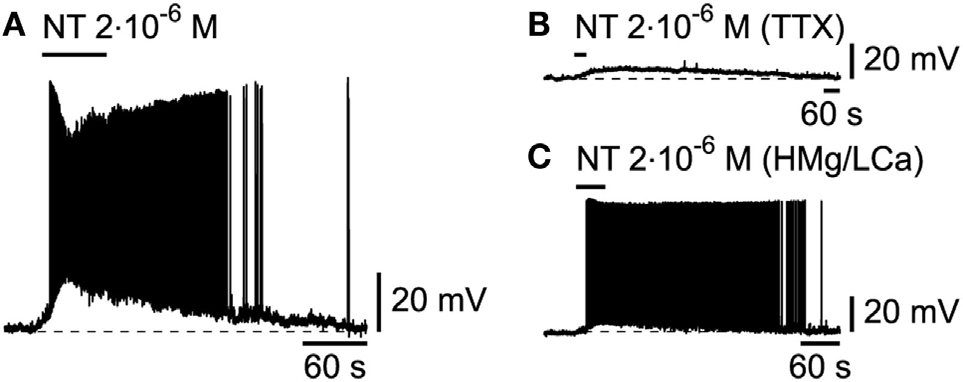
**Effect of neurotensin on L6b slow bursting neurons**. **(A)** Depolarizing and excitatory effect of neurotensin (NT), which persists in presence of TTX **(B)** or synaptic blockade **(C)**.

The recent demonstration of the colocalization of NT in hcrt/orx neurons (39), which suggests a potential role for this peptide in sleep/wake regulation, as already alluded to by its activation of basal forebrain cholinergic neurons and subsequent increase in cortical gamma activity (40), has prompted us to examine its effect on L6b bursting neurons. Similarly to other wake-promoting transmitters, NT, applied at 2.10^−6^M, produced a strong excitation (Figure 7A; *n* = 2/2) that was postsynaptic as it persisted (Figures 7B,C; *n* = 5/5) in conditions of either TTX or a high Mg/low Ca ACSF.

### Neurolucida Reconstruction of L6b Slow Bursting Neurons

**Figure 8:**
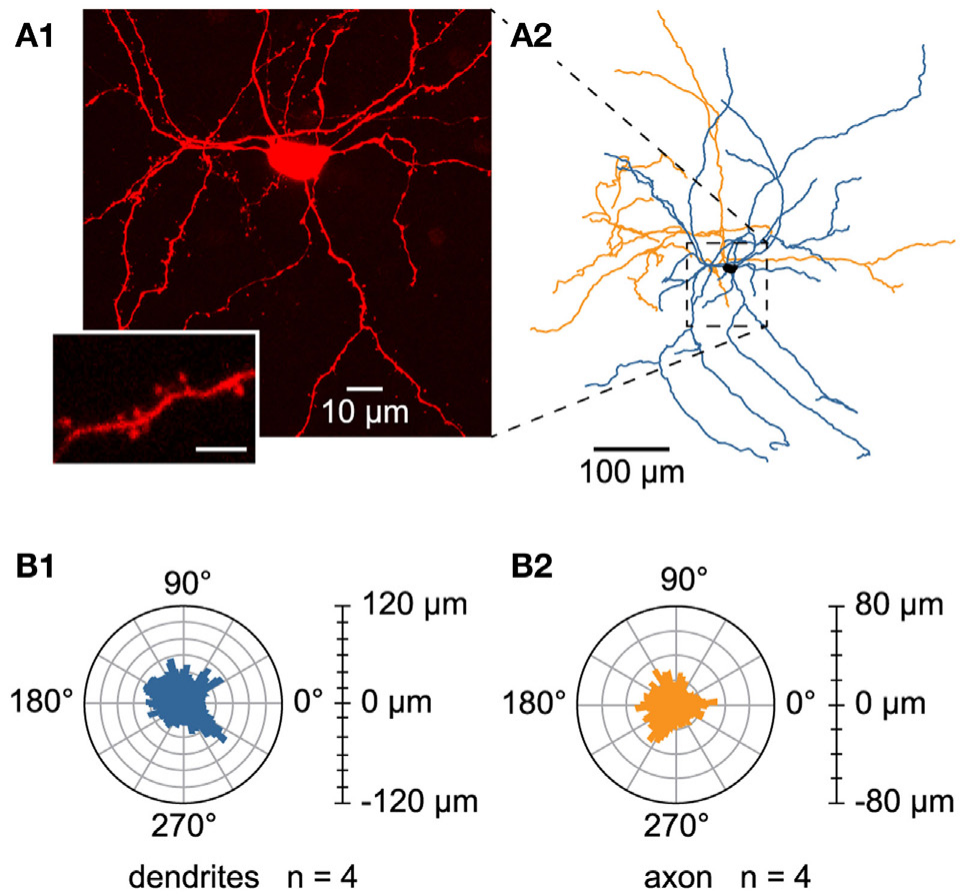
**Morphology of L6b slow bursting neurons**. **(A1)** Confocal maximum projection image obtained from an image stack of a L6b slow bursting neuron and enlargement of a spiny dendrite (lower left inset). Scale bar for the inset 2.5 μm. **(A2)** Reconstruction using Neurolucida software from the confocal image stack illustrated in A1. **(B1,B2)** Polar histograms representing the dendritic **(B1)** and axonal **(B2)** orientations of the four reconstructed cells. In each polar plot, the radius depicts the mean of the arborization length of the four reconstructed cells. The 0°–180° axis is parallel to the white matter/L6b boundary, whereas processes pointing to 90° were oriented toward the pial surface.

In order to study the morphology of dye-injected L6b slow bursting cells (Figure 8A1), Neurolucida reconstructions (Figure 8A2) and morphological analysis were performed. To minimize artifacts due to truncated arborizations, known to occur during the slicing procedure, only neurons with axon length longer than 1.5 mm were included in the analysis. This criterion was satisfied by four cells, which displayed important dendritic and axonal length (4071.5 ± 146.4 and 2359.4 ± 345.1 μm, respectively) and extended to more than 300 μm away from the soma, according to the Sholl analyses for both dendritic and axonal arborizations. The different morphological parameters characterizing these neurons are summarized in Table 1.

**Table 1:** **Morphological parameters of L6b slow bursting neurons**.

Parameters	Mean	SEM
Somatic perimeter (μm)	47.6	1.2
Somatic area (μm^2^)	134.0	6.0
Maximal feret of the soma (μm)	18.1	0.9
Minimal feret of the soma (μm)	10.9	0.9
Somatic elongation	1.7	0.2
Total dendritic length (μm)	4071.5	146.4
Number of dendritic nodes	28.0	0.7
Dendritic branching frequency (nodes/100 μm)	0.7	3.6^.^10^−2^
Dendritic length/dendritic surface	0.7	2.9^.^10^−6^
Dendritic Sholl (0–100 μm) (%)	35.7	2.9
Dendritic Sholl (100–200 μm) (%)	36.9	1.8
Dendritic Sholl (200–300 μm) (%)	20.7	2.6
Dendritic Sholl (>300 μm) (%)	6.8	2.3
Total axonal length (μm)	2359.4	345.1
Number of axonal nodes	25.3	4.8
Axonal branching frequency (nodes/100 μm)	1.1	0.1
Axonal length/axonal surface	1.4	0.2
Axonal Sholl (0–100 μm) (%)	42.7	6.0
Axonal Sholl (100–200 μm) (%)	43.2	5.0
Axonal Sholl (200–300 μm) (%)	11.1	4.1
Axonal Sholl (>300 μm) (%)	3.0	1.8

*Each parameter is given as a mean ± SEM of the cells reconstructed according to the criterion described in Section “Materials and Methods” (*n* = 4)*.

The analyzed L6b neurons had a rather ovoid soma (mean elongation 1.7 ± 0.2) with mean feret diameters of, respectively, 18.1 ± 0.9 and 10.9 ± 0.9 μm, a perimeter of 47.6 ± 1.2 μm, and a mean area of 134.0 ± 6.0 μm^2^. They appeared multipolar and spiny (see inset in Figure 8A1), with four to seven primary dendrites and no prominent apical or “apical-like” main dendrite (Figures 8A1,A2). Their dendritic branching frequency was 0.7 ± 3.6.10^−2^ nodes/100 μm and the main fraction of dendritic arborization (72.6%) was restricted to a 200-μm radius around their soma with no preferentially orientated dendrites (see the dendritic polar plot, Figure 8B1). Concerning their axonal morphology, axon collaterals arborized mainly in the same region as the dendrites with the main fraction (85.9%) located within a 200-μm radius around the cell body without any preferential orientation (see axonal polar plot, Figure 8B2). Axonal branching frequency was 1.1 ± 0.1 nodes/100 μm. It is of note that the present data could not allow us to determine the axonal projection zones of these L6b neurons within the overlying cortical layers.

## Discussion

In a previous study in rat brain slices, we have shown that, in the cerebral cortex, it is only in L6b that neurons directly sensitive to the wake-promoting transmitter hcrt/orx can be found (19). Here, in mouse cortical brain slices, we now describe, in L6b, a subset of neurons characterized by distinct intrinsic properties, a prominent propensity to discharge spontaneously in rhythmic bursts and a direct sensitivity to hcrt/orx and other transmitters of arousal. These results indicate that the action of these transmitters on these L6b cells could underlie part of their wake-promoting role.

The first issue to be discussed concerns the intrinsic electrophysiological properties of the L6b cells as described here. While their response to hyperpolarizing current steps, suggesting the presence of an *I*_h_ current, was seen in many cortical neurons, their response to depolarizing steps was rather uncommon. Indeed, with small depolarizations, one could observe a slight delay of firing, as seen in some cortical neuronal types [see, for examples, Ref. (6, 41)], but, upon stronger depolarizations, the action potentials rapidly inactivated and were replaced by a plateau potential followed by a depolarizing afterpotential. Neurons with similar properties have been described in subthalamic (42) and striatal (43) neurons of the basal ganglia, as well as in neurons of the spinal cord that belong to central pattern generators [for a recent paper, see Ref. (44)]. It is noteworthy that L6b cells with the above described properties were regularly found intermingled, during the same recording sessions, with fast spiking or regular spiking cells such as typically encountered during cortical recordings *in vitro* [for a recent paper, see Ref. (6)]. It is finally of note that some cortical GABAergic neurons expressing the neuronal nitric oxide synthase (nNOS), having also a delayed firing in response to depolarizing steps (45) and showing an increased activity during sleep (46), are also located deep in the cerebral cortex, but their possible relation to the neurons of this study remains to be explored.

The majority of L6b neurons exhibiting the intrinsic properties discussed above had a strong propensity to fire in bursts. Indeed, while a small minority of them displayed little bursting capacity, all the others were bursting either spontaneously or in response to a slight manipulation of their membrane potential. This activity was of an intrinsic origin as the bursts varied in frequency and duration in a voltage-dependent manner and were not, in most cells, subtended by synaptic activity. The contention of an intrinsic origin for this activity was further supported by the evidence that it persisted in presence of antagonists of ionotropic amino acid receptors or in a condition of full synaptic blockage. It is noteworthy that cells whose activity persisted despite amino acid receptor blockage have previously been described in layer 5 of the visual cortex (17) and layers 2–3 and 5 of the somatosensory cortex (7). Thus, they have been included in models of rhythmicity of the thalamocortical system during SWS (8). Finally, it is of interest that the firing of some L6b neurons displayed a mix of intrinsic- and network-dependent activity. This latter result, although observed only in a small minority of recordings, suggests that these L6b neurons could be embedded in a cortical network of slow rhythmic activities [for a recent study, see Ref. (6); for recent reviews, see Ref. (8, 9)].

The next issue to be discussed concerns the morphology of L6b slow bursting neurons. Neurons reconstructed in this study bear spines and are multipolar cells without any thicker dendrite or any preferential orientation. Several studies have reported the presence of multipolar morphology in L6b of rodents (27, 28, 47–49). Among these, only two quantitative studies have investigated morphological characteristics together with intrinsic membrane properties of L6b neurons specifically: one dealing with L6b GABAergic neurons of juvenile mice (49) and the other dealing with spiny, presumed glutamatergic L6b neurons of juvenile rats (48). However, the authors have not reported intrinsic electrophysiological properties such as described in this study. Our results are thus difficult to put in perspective with current literature. Differences in experimental conditions or species could explain this discrepancy.

A significant result of this study is the demonstration that these slow bursting L6b neurons are directly sensitive to hcrt/orx and other major transmitters of arousal. The evidence of a postsynaptic excitatory action of hcrt/orx on these cells extends to mice the results previously obtained in rats (19) and is in agreement with the evidence that hcrt/orx receptors are preferentially located in the deeper layers of the cerebral cortex (50). In addition, this result complements our earlier study by showing that the aminergic transmitters [reviewed in Ref. (33, 34, 51)] as well as NT, which is colocalized in hcrt/orx neurons (39) and is known to increase gamma activity (40), are all also able to activate a subset of L6b neurons in a postsynaptic manner.

Functionally, one is led to suggest that a set of L6b neurons with a propensity to fire spontaneously in slow rhythmic bursts could contribute to the SO that occur during SWS and as such be a target for wake-promoting transmitters in general and for hcrt/orx in particular [for recent reviews, see Ref. (8, 33, 34)]. Indeed, this peptide, secreted by the hcrt/orx neurons (38, 52), has been shown in recent years to play a major role in promoting waking in animals (53, 54) and humans (55, 56) as well. It would thus make sense that cortical neurons implicated in the SO present during SWS would be directly sensitive to the action of hcrt/orx, which is released at the transition from sleep to waking [for recent reviews, see Ref. (21–26)]. This transmitter will thus not only promote waking by activating, as previously shown, the arousal systems (21) and the widespread cortical-projecting midline/intralaminar nuclei (57), but also by directly impeding the ability of these L6b cortical neurons to fire in slow rhythmic bursts. In the same line, other transmitters released by hypothalamic and brainstem neurons that increase their activity at the transition between the two states [for reviews, see Ref. (33, 51)] should also directly influence the L6b cells. This is actually the case as these transmitters, known for a long time as the “classical” wake-promoting transmitters and which notably include NA, histamine, and dopamine [reviewed in Ref. (33, 34, 51)], also directly depolarize and excite the L6b slow bursting neurons. Finally, it is of interest that other layer 6b hcrt/orx sensitive neurons could possibly modulate arousal in a different manner by potentiating the action of non-specific thalamocortical inputs into layer 6a (58).

Altogether, these results lead us to speculate that, in conjunction with other neurons, notably in layer 5 (18), a set of L6b slow bursting neurons could contribute to the SO that occur during SWS and as such be a target for a direct, wake-promoting action of hcrt/orx and other transmitters associated with arousal. Ultimately, it is only *in vivo* recordings that could provide direct evidence for the precise contribution of L6b bursting neurons to the SO and SWS.

## Author Contributions

Conceived and designed the experiments: ALWC, MS, and MM. Performed the electrophysiological experiments: ALWC and MS. Performed the morphological experiments: ALWC and AD. Analyzed the data: ALWC, MS, and MM. Wrote the paper: ALWC, LB, MM, and MS.

## Conflict of Interest Statement

The authors declare that the research was conducted in the absence of any commercial or financial relationships that could be construed as a potential conflict of interest.
